# The Southern Journal of Medicine

**Published:** 1846-07

**Authors:** 


### THE SOUTHERN JOURNAL OF MEDICINE.

Favorable notices of this Journal, published at Charleston, have appeared in most of our contemporaries; we have been slow in speaking of it because we were late receiving it—the first numbers reached us only a few days since. It is edited by J. Lawrence Smith, M.D., and S. D. Sinkler, M.D., and is published bi-monthly, at $4 per annum, payable in advance. Among the contributors to its pages, are to be found Dr. Dickson, Dr. Geddings, and other professors in the Charleston Medical College. The three numbers before us contain some very able original papers, and their whole appearance indicates, that the Journal is to be one of a high order. There is no reason in the world why Charleston, once the literary emporium of America, should not sustain such a journal; and it is perfectly clear that the profession at the South, if it step not forth heartily in support of such an enterprise, will do itself great injustice.

